# Seeing to hear? Patterns of gaze to speaking faces in children with autism spectrum disorders

**DOI:** 10.3389/fpsyg.2014.00397

**Published:** 2014-05-08

**Authors:** Julia R. Irwin, Lawrence Brancazio

**Affiliations:** ^1^Haskins LaboratoriesNew Haven, CT, USA; ^2^Department of Psychology, Southern Connecticut State UniversityNew Haven, CT, USA

**Keywords:** autism spectrum disorders, audiovisual speech perception, eyetracking, communication development, speech in noise, lipreading

## Abstract

Using eye-tracking methodology, gaze to a speaking face was compared in a group of children with autism spectrum disorders (ASD) and a group with typical development (TD). Patterns of gaze were observed under three conditions: audiovisual (AV) speech in auditory noise, visual only speech and an AV non-face, non-speech control. Children with ASD looked less to the face of the speaker and fixated less on the speakers’ mouth than TD controls. No differences in gaze were reported for the non-face, non-speech control task. Since the mouth holds much of the articulatory information available on the face, these findings suggest that children with ASD may have reduced access to critical linguistic information. This reduced access to visible articulatory information could be a contributor to the communication and language problems exhibited by children with ASD.

## INTRODUCTION

Autism spectrum disorders (ASD) refer to neurodevelopmental disorders along a continuum of severity that are generally characterized by marked deficits in social and communicative functioning (American Psychiatric Association, 2000). A feature of the social deficits associated with ASD is facial gaze avoidance and reduced eye contact with others in social situations (Hutt and Ounstead, 1966; Hobson et al., 1988; Volkmar et al., 1989; Volkmar and Mayes, 1990; Phillips et al., 1992). One implication of this reduced gaze to other’s faces is a potential difference in face processing. A number of studies have suggested that individuals with ASD show differences in face processing, including impaired face discrimination and recognition (for a review see Dawson et al., 2005, but see Jemel et al., 2006 for evidence that face processing abilities are stronger in ASD than previously reported) and identification of emotion (Pelphrey et al., 2002).

Along with identity and affective information, the face provides valuable information about a talker’s articulations. Visible speech information influences what typically developing listeners hear (e.g., increases identification in the presence of auditory noise, Sumby and Pollack, 1954) and is known to facilitate language processing (McGurk and MacDonald, 1976; MacDonald and McGurk, 1978; Reisberg et al., 1987; Desjardins et al., 1997; MacDonald et al., 2000; Lachs and Pisoni, 2004). Further, typical speech and language development is thought to take place in an audiovisual (AV) context (Meltzoff and Kuhl, 1994; Desjardins et al., 1997; Lachs et al., 2001; Bergeson and Pisoni, 2004). Thus, differences in access to visible speech information would have significant consequences for a perceiver. For example, there is evidence that the production of speech differs in blind versus sighted individuals (for example, sighted speakers produce vowels further apart in articulatory space than those of blind speakers, ostensibly because of their access to visible contrasts; Menard et al., 2009), suggesting that speech perception and production is influenced by experience with the speaking face.

Consistent with their difficulties with information on faces, a growing body of literature indicates that children with ASD are less influenced by visible speech information than TD controls (De Gelder et al., 1991; Massaro and Bosseler, 2003; Williams et al., 2004; Mongillo et al., 2008; Iarocci et al., 2010; Irwin et al., 2011, but see Iarocci and McDonald, 2006 and Woynaroski et al., 2013). In particular, children and adolescents with ASD appear to benefit less from the visible articulatory information on the speaker’s face in the context of auditory noise (Smith and Bennetto, 2007; Irwin et al., 2011). Further, children with ASD have been reported to be particularly poor at lipreading (Massaro and Bosseler, 2003).

Although avoidance of gaze to others’ faces has been noted clinically, the exact nature of gaze patterns to faces in ASD has been a topic of investigation. A varied body of research using eye-tracking methodology has examined patterns of facial gaze patterns in individuals with ASD, in particular with complex social situations and with affective stimuli. A number of studies find that individuals with ASD differ in the amount of fixations to the eye region of the face when compared to typically developing (TD) controls (Klin et al., 2002; Pelphrey et al., 2002; Dalton et al., 2005; Boraston and Blakemore, 2007; Speer et al., 2007; Kleinhans et al., 2008; Sterling et al., 2008). In particular, during affective or emotion based tasks, individuals with ASD have been reported to spend significantly more time looking at the mouth (Klin et al., 2002; Neumann et al., 2006; Spezio et al., 2007). However, a recent review by Falck-Ytter and von Hofsten (2011) calls into question whether individuals with ASD look less to the eyes and more to the mouth when gazing at faces; they argue that only limited support exists for this in adults and even less evidence in children. Apart from gaze to eyes and mouth, some studies show increased gaze at “non-core” features (e.g., regions other than the eyes, nose, and mouth) of the face by individuals with ASD compared to TD controls, when gazing at facial expression of emotion (Pelphrey et al., 2002). Reports of differences in patterns of gaze to faces are not unequivocal, however, with a number of studies reporting no group differences in certain tasks (Adolphs et al., 2001; Speer et al., 2007; Kleinhans et al., 2008). Further, when assessing gaze to a face, pattern of gaze may be a function of both language skill and development. Norbury et al. (2009) report that pattern of gaze to the mouth is associated with communicative competence in ASD. Reported differences in gaze to faces in children with ASD appear to vary depending on the age of the child (Dawson et al., 2005; Chawarska and Shic, 2009; Senju and Johnson, 2009). Moreover, recent work by Foxe et al. (2013) suggests that multisensory integration deficits present in children with ASD may resolve in adulthood (although subtle differences may persist; Saalasti et al., 2012).

Critically, little is known about gaze to the face during speech perception tasks. A question that arises is whether the previously reported deficit in visual speech processing in children with ASD might simply be a consequence of a failure to fixate on the face. However, recent findings by Irwin et al. (2011) provide evidence against this possibility. Irwin et al. (2011) tested children with ASD and matched TD peers on a set of AV speech perception tasks while concurrently recording eye fixation patterns. The tasks included a speech-in-noise task with auditory-only (static face) and AV syllables (to measure the improvement in perceptual identification with the addition of visual information), a McGurk task (with mismatched auditory and visual stimuli), and a visual-only (speechreading) task. Crucially, Irwin et al. (2011) excluded all trials where the participant did not fixate on the speaker’s face. They found that even when fixated on the speaker’s face, children with ASD were less influenced by visible articulatory information than their TD peers, both in the speech-in-noise tasks and with AV mismatched (McGurk) stimuli. Moreover, the children with ASD were less accurate at identifying visual-only syllables than the TD peers (although their overall speechreading accuracy was fairly high).

Irwin et al.’s (2011) findings indicate that fixation on the face is not sufficient to support efficient AV speech perception. This could suggest differences in how visual speech information is processed in individuals with ASD. However, it could also be due to different gaze patterns on a face exhibited by individuals with ASD. Perhaps if they tend to fixate on different regions of the face than TD individuals, individuals with ASD have reduced access to critical visual information. Consistent with this possibility is evidence that attentional factors can modulate visual influences in speech perception in typical adults; visual influence is reduced when perceivers are asked to attend to a distractor stimulus on the speaker’s face (Alsius et al., 2005). Typically developing adults have been shown to increase gaze to the mouth area of the speaker as intelligibility decreases during AV speech tasks (Yi et al., 2013). Further, Buchan et al. (2007) report that typically developing adults gaze to a central area on the face in the presence of AV speech in noise, reducing the frequency of gaze fixations on the eyes and increasing gaze fixations to the nose and the mouth. If children with ASD do not have access to the same visible articulatory information as the TD controls because their gaze patterns differ, this may influence their perception of a speaker’s message.

To assess whether there are differences in gaze that underlie the AV speech perception differences in children with ASD as compared to children with typical development, for the present paper we conducted a detailed analysis of the eye-gaze patterns for the participants and tasks reported in Irwin et al. (2011). In particular, we examined patterns of gaze to a speaking face under perceptual conditions where there is an incentive to look at the face: (1) in the presence of auditory noise and (2) where no auditory signal is present (speechreading). We tested whether children with ASD differ from TD controls not only in overall time spent on the face, but also in the relative amount of time spent fixating on the mouth and non-focal regions. We further examined whether the two groups differ in the time-course of eye-gaze patterns to these regions over the course of a speech syllable. Given that the children with ASD in this sample exhibited poorer use of visual speech information than the TD controls in perceptual measures (both for visual-only and AV speech), the analyses reported here may shed some light on the basis for these differences: Is reduced use of visual speech information in perception associated with differences in patterns of fixation on the talking face?

Finally, as a control for the possibility that there are more general group differences in gaze pattern unrelated to faces, we also analyzed gaze patterns in a control condition with dynamic AV non-face, non-speech stimuli.

## MATERIALS AND METHODS

### PARTICIPANTS

Participants in the current study were 20 native English speaking monolingual children, 10 with ASD (eight boys, mean age 10.2 years, age range 5.58–15.9 years) and 10 TD controls (eight boys, mean age 9.6, age range 7–12.6 years). Because the speech conditions in this study required the child participants to report what the speaker said, all participants in this study were verbal. All child participants were reported by parents to have normal or corrected-to-normal hearing and vision. The TD participants had no history of developmental delays including vision, hearing, speech or language problems, by parent report.

The TD controls were matched with the child ASD participants on sex, age, cognitive functioning and language skill. The TD controls were taken from a larger set of children participating in a study of speech perception (*n* = 80). In addition, the primary caregivers of children with ASD completed a diagnostic interview [autism diagnostic interview-revised (ADI-R), Lord et al., 1994] about their children (*n* = 10 adult females).

Prior to their participation in the study, child participants with ASD received a diagnosis from a licensed clinician. Four participants had a diagnosis of autism, four of Asperger syndrome and two were diagnosed with pervasive developmental disorder not otherwise specified (PDD-NOS); these diagnoses all fall within the classification of ASD. For characterization purposes, participants with ASD were also assessed with the autism diagnostic observation schedule (ADOS; Lord et al., 2000), and their caregivers (*n* = 10) were interviewed with the ADI-R (Lord et al., 1994). All participants with ASD met or exceeded cutoff scores for autism spectrum or autism proper on the ADOS algorithm. Scores obtained from caregiver interviews showed that the children with ASD met or exceeded cutoff criteria on the language/communication, reciprocal social interactions and repetitive behavior/interest domains on the ADI-R. Consistent with the range of clinical diagnoses, there was heterogeneity in the extent of social and communication deficits and presence of restricted and repetitive behavior (for example, scores on the combined communication and social impairment scales in the ADOS ranged from 7 to 20, where 10 is the minimum cutoff score and 22 is the maximum possible score).

The mean age and standard deviations of the child ASD and child TD participants, along with measures of cognitive and language functioning, are presented in **Table 1**. The measures of cognitive functioning were standardized scores for general conceptual ability (GCA) on the Differential Abilities Scale (DAS); the measures of language function were core language index scores (CLI) from the clinical evaluation of language fundamentals-4 (CELF-4; Semel et al., 2003). Independent-samples *t*-tests on age, GCA, and CLI did not reveal significant differences between the groups, as shown in **Table 1**.

**Table 1:** Mean age and cognitive and language measures for the children with ASD and TD.

	ASD	TD	*T*-test
*n*	10	10	
Age	10.2 (3.1)	9.6 (2.4)	*t*(18) = -0.51, ns
General conceptual ability (GCA)	92.1 (15.5)	98.9 (15.5)	*t*(18) = 0.97, ns
Core language index scores (CLI)	87.4 (17.3)	97.8 (15.1)	*t*(18) = 1.4, ns

*GCA and CLI are standardized scores. Standard deviations are in parentheses.*

The sample included here represents a subset of the participants whose data were reported in Irwin et al. (2011). The data of three children with ASD and one TD control were excluded from the present analyses because they spent too little time fixating on the face to permit statistical analysis. The data of two other TD control participants were also removed due to the removal of their respective matched ASD participants.

### MATERIALS

#### Stimuli

***Speech stimuli.*** The speech stimuli were created from a recording of the productions of a male, monolingual, native speaker of American English. This speaker was audio- and video-recorded in a recording booth producing a randomized list of the consonant-vowel (CV) syllables /ma/ and /na/. The video was centered on the speaker’s face and was framed from just above the top of the speaker’s head to just below his chin, and was captured at 640 × 480 pixels. The audio was simultaneously recorded to computer and normalized for amplitude, and then realigned with the video in Final Cut Pro. Two tokens of /ma/ and /na/ were selected as stimuli. The stimuli were trimmed to start with the mouth position at rest, followed by an opening gesture, closing for the consonant, and release of the consonant into the following vowel, and ended with the mouth returning to rest at the end of the syllable. The stimuli were approximately 1500 ms long, with the acoustic onset of the consonant (for the AV stimuli) occurring at around 600 ms; the acoustic portions of the stimuli were approximately 550 ms in duration, on average.

For AV speech in noise, the stimuli were AV stimuli of /ma/ and /na/. Three versions of each stimulus was created by setting the mean dB of the syllables at 60 dBA, and then adding pink noise at 70, 75, and 70 dBA to the AV /ma/ and /na/ tokens to create stimuli with a range of signal-to-noise levels from less to more noisy (i.e., -10, -15, and -20 dB S/N, respectively). Noise onset and offset were aligned to the auditory speech syllable onset and offset.

The visual-only (speechreading) stimuli were identical to the AV stimuli, except that the audio channel was removed.

**FIGURE 1:**
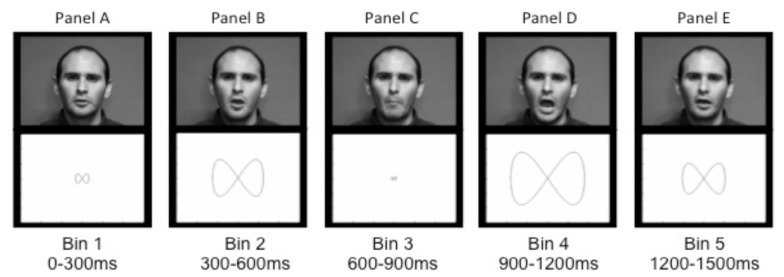
**Sample images of the speaker (top panels) during a production of /ma/ and the corresponding non-speech figure-eight shapes (lower panels) taken from each time bin.** Panels A through E illustrate, respectively, the initial rest position **(A)**, opening prior to the consonant closing gesture **(B)**, the closure for /m/ **(C)**, peak mouth opening for the vowel **(D)**, and the return to rest at the end of the vowel **(E)**.

***Non-speech control stimuli.*** The AV non-speech stimuli consisted of a set of figure-eight shapes that increased and decreased in size, paired with sine-wave tones that varied in frequency and amplitude. These stimuli were modeled on the speaker’s productions of /ma/ and /na/ but did not look or sound like speech. To create the visual stimulus, we measured the lip aperture in every video frame of the /ma/ and /na/ syllables. We then used the aperture values to drive the size of the figure: when the lips closed the figure was small, and upon consonant release into the vowel the figure expanded (see **Figures 1C,D**). The auditory stimuli were created by converting the auditory /ma/ and /na/ syllables into sine-wave analogs, which consist of three or four time-varying sinusoids, following the center-frequency and amplitude pattern of the spectral peaks of an utterance (Remez et al., 1981). These sine-wave analogs sound like chirps or tones. Thus, the AV non-speech stimuli retained the temporal dynamics of speech, without looking or sounding like a speaking face (see **Figures 1A–E**).

#### Visual tracking methodology

Visual tracking was done with an ASL Model 504 pan/tilt remote tracking system, a remote video-based single eye tracker that uses bright pupil, coaxial illumination to track both pupil and corneal reflections at 120 Hz. To optimize the accuracy of the pupil coordinates obtained by the optical camera, this model has a magnetic head tracking unit that tracks the position of a small magnetic sensor attached to the head of the participant, above their left eye.

#### Language assessment

Language ability was assessed with the CELF-4 (Semel et al., 2003). The CELF-4 is reliable in assessing the language skills of children in the general population and those with a clinical diagnosis including ASD (Semel et al., 2003).

#### Cognitive assessment

Cognitive ability was assessed using the Differential Ability Scales (DAS) School Age Cognitive Battery (Elliott, 1991). The DAS provides a GCA score, which assesses verbal ability, non-verbal reasoning ability, and spatial ability.

#### ADOS

Children with ASD were assessed with the ADOS generic (ADOS-G). The ADOS is a semi-structured standardized assessment of communication, social interaction, and play/imaginative use of materials for individuals suspected of having an ASD (Lord et al., 2002).

#### ADI-R

Caregivers of participants with ASD were given the ADI-R (Lord et al., 1994). The ADI-R is a standardized, semi-structured interview for caregivers of those with an ASD to assess autism symptomatology.

### PROCEDURE

After consent was obtained in accordance with the Yale University School of Medicine, all participants completed the experimental tasks in the eye-tracker. Each participant was placed in front of the monitor, after which calibration of the participant’s fixation points in the eye-tracker was completed. Prior to any stimulus presentation for each task, directions appeared on the monitor. These directions were read aloud to the participant by a researcher to ensure that they understood the task. In addition, two practice items were completed with the researcher present to confirm that the participant understood and could complete the task. For all conditions, if participants were unsure, they were asked to guess.

#### Condition 1: AV speech in noise

Participants were told that they would see and hear a man saying some sounds that were not words and to say out loud what they heard. Each of the six stimuli (two different tokens of each /ma/ and /na/, at each of the three levels of signal-to-noise ratios) was presented four times, for a total of 24 trials in a random sequence.

#### Condition 2: visual only (speechreading)

Participants were told that they would see a man saying some sounds that they would not be able to hear, and then asked to say out loud what they thought the man was saying. Each of the four stimuli (two different tokens of each /ma/ and /na) was presented five times, for a total of 20 trials in a random sequence.

#### Condition 3: non-speech control

For this task, two stimuli were presented in sequence on each trial. The paired stimuli were either modeled on different tokens of the same syllable (e.g., both /ma/ or both /na/) or on tokens of different syllables (one /ma/ and one /na/). Participants were told that they would see two shapes that would open and close and should say out loud whether the two shapes opened and closed in the same way (e.g., both modeled on /ma/ or both modeled on /na/, although no reference was made to the speech origins of the stimuli to participants) or if the way that they closed was different (e.g., one modeled on /ma/ and one on /na/). Each pairing was presented seven times, for a total of 28 trials in a random sequence.

The three tasks were blocked and presented in random order. The inter-stimulus interval for all trials within the blocks was 3 s. After every five trials, participants were presented with a slide of animated shapes and faces, to maintain attention to the task. All audio stimuli were presented at a comfortable listening level (60 dBA) from a centrally located speaker under the eye-tracker, and visual stimuli were presented at a 640 × 480 aspect ratio on a video monitor 30 inches from the participant.

After the experimental procedure participants were tested with the battery of cognitive and language assessments and caregivers of the ASD participants were interviewed separately with the ADI-R.

## RESULTS

Participant gaze to the speaker’s face was examined by group for the AV speech-in-noise and visual-only (speechreading) trials, as was gaze on the figure-eight shape in non-speech trials. The eye tracker recorded fixation position in x and y coordinates at approximately 8 ms intervals. (In cases where the coordinates were not recorded, the x- and y-coordinates of the previous time point were applied). Each x-y coordinate was coded according to whether it was on-screen or off-screen, and if it was on-screen, whether it was part of an on-face fixation or not. Off-screen fixations were eliminated from the data.

The on-face coordinates were coded according to face regions, namely: forehead, jaw, cheeks, ears, eyes, mouth region (including the spaces between the lower lip and the jaw and between the upper lip and the nose), and nose. The primary regions of interest were the *mouth region* and a collective set of *non-focal regions* (face areas other than the mouth region, eyes, and nose), in light of reports that children with ASD spend relatively more time fixating on non-focal regions of the face (Pelphrey et al., 2002). The non-focal regions encompassed the ears, the cheeks, the forehead, and all other regions not otherwise labeled (primarily the space between the eye and the ear, between the nose and cheek, and between the eyes). The jaw area was not included in either the mouth region or the non-focal regions; this is because the jaw, unlike the other non-focal regions, has extensive movement that is time-locked to the speech articulation – thus, jaw movement conveys information about the kinematics of the speech act.

For the non-speech condition, the on-screen regions were coded in an analogous manner, based on the extent of the figure-eight shape. These regions are described below.

Data points were only included as fixations if they had less than a 40 pixel movement from the previous time point, and occurred within a contiguous 100 ms window of similar small movements that did not cross into a different face region, as defined above. In all, 14.5% of the time steps were eliminated across the AV speech-in-noise and visual-only tasks for being either off-screen, saccades, or blinks. Although the mean percentage of dropped data points was higher for the ASD sample than for the TD sample, the difference was not statistically significant [for AV speech-in-noise, ASD: *M* = 19.4%, SD = 13.3; TD: *M* = 11.8%, SD = 7.4; *t*(18) = 1.60, ns; for visual-only, ASD: *M* = 17.0%, SD = 12.0; TD: *M* = 10.0%, SD = 5.3; *t*(18) = 1.70, ns].

The individual time steps were collapsed into 300 ms time bins (0–300 ms, 300–600 ms, 600–900 ms, 900–1200 ms, and 1200–1500 ms); we thus calculated the total amount of time spent in each region within each time bin. These time bin boundaries were selected because they roughly corresponded to visual landmarks in the speech signal. The first bin (0–300 ms) preceded the onset of visible movement; the second bin (300–600 ms) included opening of the mouth prior to the consonant and the initiation of closing (either lips in /ma/ or upward tongue-tip movement in /na/); the third bin (600–900 ms) included the consonantal closure and release, and the final two time bins (900–1200 ms and 1200–1500 ms, respectively) span production of the vowel until the end of the trial (for an image of articulation in each of the time bins paired with the corresponding figure-eight shape, see **Figure 1**).

As a result, our dependent variables were the mean percentage of time gazing on a given region within a time bin. Time spent fixating on the *face* was calculated as a percentage of time fixated anywhere on the computer monitor within each time bin. In contrast, time spent fixating on *specific face regions* (mouth region and non-focal areas) was calculated as a percentage of time spent fixated on the face within each time bin.

**FIGURE 2:**
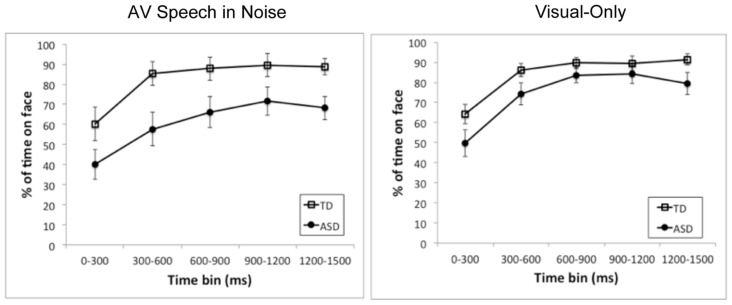
**Mean time spent on the face region as a percentage of time spent on-screen for each of the time bins and for the ASD group (closed circles) and the TD group (open squares).** The left and right panels present results for AV speech in noise and visual-only, respectively. Error bars represent standard errors, calculated independently for each time bin.

First, we examined whether there were group differences in the percentage of time spent fixating on the *face* of the speaker out of time spent fixating on-screen. **Figure 2** presents the mean time spent on face by group and time bin separately for the AV speech-in-noise and visual-only tasks. As the figure shows, the ASD group on average spent consistently less time on the face than the TD group in both tasks. A set of 2 (group: ASD, TD) by 5 (time bin: 0–300 ms, 300–600 ms, 600–900 ms, 900–1200 ms, and 1200–1500 ms) mixed factor analyses of variance (ANOVAs) were conducted for AV speech-in-noise and visual-only, respectively. There was a significant main effect of group with less time spent on the face by the ASD group than the TD group for AV speech-in-noise with a marginal effect for visual-only [for AV speech-in-noise, ASD: *M* = 60.8, SD = 25.0; TD: *M* = 82.3, SD = 21.9; *F*(1,18) = 6.31, *p* = 0.02, $\eta_{G}^{2}$ = 0.22; for visual-only, ASD: *M* = 74.3, SD = 20.7; TD: *M* = 84.2, SD = 14.9; *F*(1,18) = 3.39, *p* = 0.08, $\eta_{G}^{2}$ = 0.12]. These mean differences reflect moderate to large effect size estimates (Cohen, 1973; Olejnik and Algina, 2003; Bakeman, 2005). There was also a main effect of time bin in both analyses [AV speech-in-noise: *F*(4,72) = 26.48, *p* < 0.0001, $\eta_{G}^{2}$ = 0.23; visual-only: *F*(4,72) = 42.7, *p* < 0.001, $\eta_{G}^{2}$ = 0.41], reflecting a rapid increase in fixations on the face from the first to second bins that leveled off by the third bin. The interaction of group and time was not significant for either task.

**FIGURE 3:**
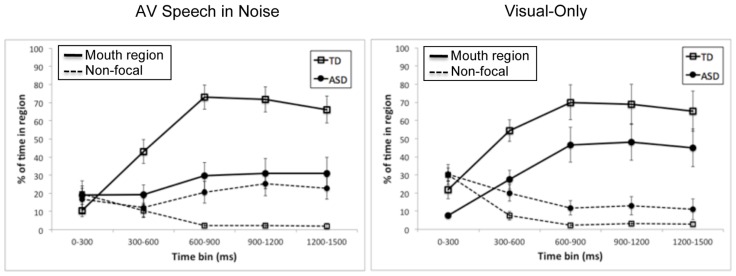
**Mean time spent on the mouth region (solid lines) and non-focal areas (dashed lines) as a percentage of time spent on the face for each of the time bins and for the ASD group (closed circles) and the TD group (open squares).** The left and right panels present results for AV speech in noise and visual-only, respectively. Error bars represent standard errors, calculated independently for each time bin.

Next, we examined whether there were group differences in gaze to specific regions on the face. We chose the mouth region and non-focal areas (as defined above) as regions of interest^1^. We ran a set of 2 (group: ASD, TD) by 5 (time bin: 0–300 ms, 300–600 ms, 600–1200 ms, 1200–1500 ms) ANOVAs on the percentage of time spent in each region of interest out of time spent on the face, with separate analyses for the AV speech-in-noise and visual-only tasks, and separate analyses for the mouth region and non-focal areas. **Figure 3** presents the relative percentages of time spent in each region of interest by group and time, separately for the AV speech-in-noise and visual-only tasks.

First, consider the *mouth region*. There was a significant main effect of group for both tasks, with a relatively smaller percentage of time spent on the mouth region for the ASD group than the TD group [for AV speech-in-noise, ASD: *M* = 26.0, SD = 24.1; TD: *M* = 52.9, SD = 30.8; *F*(1,18) = 11.25, *p* < 0.005, $\eta_{G}^{2}$ = 0.29; for visual-only, ASD: *M* = 35.0, SD = 29.5; TD: *M* = 56.1, SD = 32.6; *F*(1,18) = 4.46, *p* = 0.05, $\eta_{G}^{2}$ = 0.14]. There was also a main effect of time for both tasks [AV speech-in-noise: *F*(4,72) = 23.18, *p* < 0.0001, $\eta_{G}^{2}$ = 0.32; visual-only: *F*(4,72) = 23.7, *p* < 0.0001, $\eta_{G}^{2}$ = 0.30], with an overall increase in fixations on the mouth region from the first to third bins before leveling off. Interestingly, there was an interaction of group and time bin for AV speech-in-noise [*F*(4,72) = 10.06, *p* < 0.0001, $\eta_{G}^{2}$ = 0.17], but not for visual-only (*F* < 1). As shown in **Figure 3**, for AV speech-in-noise, fixations on the mouth region were similar for the two groups in the first time bin (0–300 ms, prior to the onset of mouth movement), but the subsequent increase in mouth region fixations was much more pronounced for the TD group than the ASD group. In contrast, in the visual-only task the two groups’ trajectories across time were similar, differing in overall percentage of time in the mouth region.

Next, consider the *non-focal regions*. For AV speech-in-noise, there was a significant main effect of group, with a relatively higher percentage of time spent fixating on non-focal regions by the ASD group than the TD group [ASD: *M* = 19.5, SD = 19.6; TD: *M* = 7.3, SD = 10.5; *F*(1,18) = 6.48, *p* < 0.05, $\eta_{G}^{2}$ = 0.15]. There was not a significant main effect of time, *F*(4,72) = 1.11, ns, but there was a significant interaction of group and time, *F*(4,72) = 4.98, *p* < 0.005, $\eta_{G}^{2}$ = 0.12. Time spent on non-focal regions was similar for the two groups in the first time bin, but dropped off rapidly for the TD group while remaining relatively frequent for the ASD group across the whole trial. For visual-only, there was again a main effect of group [ASD: *M* = 17.3, SD = 16.9; TD: *M* = 9.2, SD = 12.6; *F*(1,18) = 5.43, *p* < 0.05, $\eta_{G}^{2}$ = 0.11], along with a significant main effect of time, *F*(4,72) = 17.64, *p* < 0.0001, $\eta_{G}^{2}$ = 0.37, with a decrease in time spent on non-focal regions from the first time bin to the subsequent bins. The interaction of group and time (*F* < 1) was not statistically significant in the visual-only task^2^.

The results in the speech tasks can be summarized as follows. First, the ASD group spent, on average, less time gazing on the face than the TD group, and this difference was more pronounced in the AV speech-in-noise task than in the visual-only task. Second, when fixating on the face, the ASD group spent relatively less time fixating on the mouth region than the TD group, and relatively more time fixating on non-focal regions. Finally, the two groups differed in their relative pattern of fixations on the speech over the course of a trial. Specifically, the TD group exhibited a pattern of initially looking at non-focal regions but then shifting to the mouth as the articulation unfolded. The ASD group had a similar but reduced shift in the visual-only task, but did not exhibit this shift in the AV speech-in-noise task.

### NON-SPEECH CONTROL CONDITIONS

**FIGURE 4:**
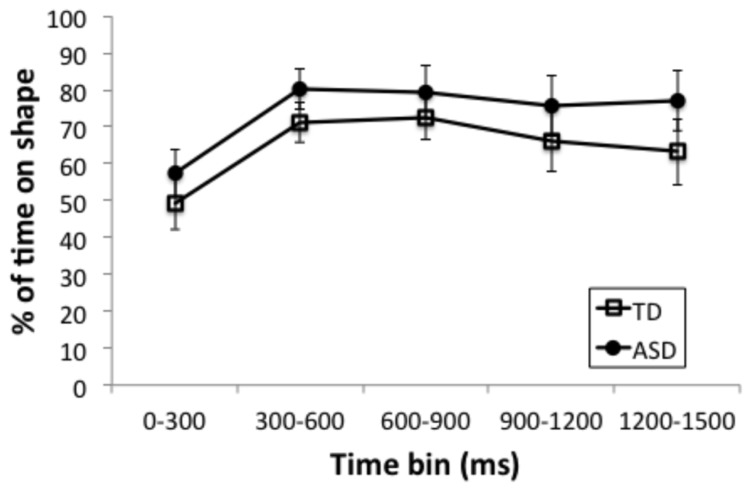
**Mean time spent on the figure-eight shape region as a percentage of time spent on-screen for each of the time bins and for the ASD group (closed circles) and the TD group (open squares).** Error bars represent standard errors, calculated independently for each time bin.

Finally, to assess whether there were group differences in gaze to the non-speech stimuli, a series of independent 2 (group: ASD, TD) × 5 (time bins: 0–300 ms, 300–600 ms, 600–900 ms, 900–1200 ms, and 1200–1500 ms) ANOVAs were run on fixations to the figure-eight shapes during time spent on screen. The earliest time bin encompasses pre-movement (0–300 ms), the next time bin (300–600 ms) an increase to maximum size; the third time bin (600–900 ms) from maximum size to minimum size and the final two time bins increasing until the end of the trial (900–1200 ms, 1200–1500 ms, see **Figure 1**). We defined two regions of interest: a narrow region encompassing an area around the outline of the figure-eight shape at its smallest point (see **Figure 1C**), and a broad region encompassing the area around the outline of the shape at its largest point (see **Figure 1D**). We analyzed percentage of trials with fixations in each region at the previously defined time samples that incorporated the shape’s transition from a small outline to a large one. The percentage of time spent in the broad region, shown in **Figure 4**, had a main effect of time bin [*F*(4,72) = 12.33, *p* < 0.0001, $\eta_{G}^{2}$ = 0.13], due to an increase from the first bin (prior to movement) to the second, but no main effect of group [*F*(1,18) = 1.09, ns] and no interaction of group and time bin (*F* < 1). The percentage of time in the narrow region also had a main effect of time bin [*F*(4,72) = 8.32, *p* < 0.001, $\eta_{G}^{2}$ = 0.14], with less time in the inner region in the first bin (prior to movement) and in the last two bins (when the shape was larger), but again with no main effect of group (*F* < 1) and no interaction of group and time bin [*F*(4,72) = 1.10, ns]. Overall, the TD and ASD groups exhibited similar gaze patterns with the non-speech stimuli.

## DISCUSSION

The current study examined pattern of gaze to a speaking face by children with ASD and a set of well-matched TD controls. Gaze was examined under conditions that create a strong incentive to attend to the speaker’s articulations, namely, AV speech with background noise and visual only (speechread) speech. We found differences in the gaze patterns of children with ASD relative to their TD peers, which could impact their ability to obtain visible articulatory information.

The findings indicated that children with ASD spent significantly less time gazing to a speaking face than the TD controls, which is consistent with diagnostic criteria for this disorder and findings from previous research (Hutt and Ounstead, 1966; Hobson et al., 1988; Volkmar et al., 1989; Volkmar and Mayes, 1990; Phillips et al., 1992). The reduction in gaze to the face of the speaker was greater in the speech in noise than the visual-only condition. This suggests that children with ASD gaze at the face of the speaker when the task requires it, as in speechreading. This is perhaps consistent with the finding that the difference in perceptual performance between the ASD and TD groups (Irwin et al., 2011) was less pronounced in the visual-only condition than with speech in noise.

Importantly, when fixated on the face of speaker, the children with ASD were significantly less likely to gaze at the speaker’s mouth than the TD children in the context of both speech in noise and speechreading. This finding might appear to conflict with previous findings of increased gaze to the mouth by individuals with ASD in comparison to TD controls (e.g., Klin et al., 2002; Neumann et al., 2006; Spezio et al., 2007). However, this disparity may arise from the specific demands of the respective tasks. Findings of increased gaze on the mouth by children with ASD have typically occurred when the task required emotional or social judgments and when the mouth was not the primary source of the relevant information. In contrast, our study involved a speech perception task, so the mouth *was* the primary source of relevant (articulatory) information. These findings in tandem suggest that children with ASD paradoxically may be *less* likely to attend to the mouth when it carries *greater* informational value.

Instead of gazing at the mouth during the speech in noise task, the children with ASD tended to spend more time directing their gaze to non-focal areas of the face (also see Pelphrey et al., 2002). Non-focal areas such as the ears, cheeks, and forehead carry little, if any, articulatory information. For speech in noise, as the speaker began to produce the articulatory signal, the TD children looked more to the mouth than did the children with ASD, who continued to gaze at non-focal regions.

Notably, the group differences were less prominent in the visual-only condition, where visual phonetic information on the mouth is fundamental to the task (in contrast to the speech-in-noise task, where there is an auditory speech signal). In this case, the two groups exhibited a similar pattern of shifting from non-focal areas to the mouth region as the speaker began to produce the syllable, even though the ASD group overall spent relatively less time on the mouth and more time on non-focal regions than the TD controls. This finding suggests that children with ASD may be able to approximate a similar pattern of gaze to areas of the face that hold important articulatory information when it is required by the task.

Finally, there were no significant differences by group in pattern of gaze for the non-speech, non-face control condition. This suggests that the differences in gaze patterns between children with ASD and TD do not necessarily occur for all AV stimuli, and are consistent with the notion that these differences are specific to speaking faces.

In the Introduction, we outlined two possible reasons for why children with ASD are less influenced by visual speech information than their TD peers, even when they are fixated on the face (Irwin et al., 2011), namely, that they have an impairment in AV speech processing, or that they have reduced access to critical visual information. The present results do not address the question of a processing impairment, but they do offer insight into the issue of access to speech information. Because the mouth is the source of phonetically relevant articulatory information available on the face (Thomas and Jordan, 2004), our results may help account for the language and communication difficulties exhibited by children with ASD.

To summarize, even with a sample of verbal children who were closely matched in language and cognition to controls, we found differences in pattern of gaze to a speaking face between children with ASD and TD controls. However, these findings should be interpreted with caution, given the small sample size, broad age range and varied diagnostic category. Future research should be conducted to assess how differences in each of these variables impacts pattern of gaze. In particular, an interesting question is whether pattern of gaze relates to communicative skill (e.g., as in Norbury et al., 2009; also see Falck-Ytter et al., 2012). A larger sample would allow for examination of this relationship. Further, the speech stimuli in the current study were consonant-vowel speech syllables; future research should also examine sentence level connected speech.

Finally, future work should consider the possible implications of the results for intervention. Our results in the speech-in-noise task indicate that children with ASD may not spontaneously look to critical areas of a speaking face in the presence of background noise, even though it would improve comprehension. This is particularly problematic in light of findings that auditory noise is especially disruptive for individuals with ASD in speech perception (Alcántara et al., 2004). However, the results in the visual-only speechreading task, where children with ASD did tend to shift their gaze from non-focal areas to the mouth (albeit to a lesser degree than the TD controls), suggests that children with ASD can show more typical gaze patterns when necessary. Therefore, intervention to help individuals with ASD to gain greater access to visible articulatory information may be useful, with the goal of increased communicative functioning in the natural listening and speaking environment.

## Conflict of Interest Statement

The authors declare that the research was conducted in the absence of any commercial or financial relationships that could be construed as a potential conflict of interest.

## Footnotes

1 In addition to the analyses of the mouth region and non-focal regions, we also conducted statistical analyses of fixations on other major face areas, namely the eyes and nose. However, each involved few fixations overall and the analyses did not reveal reliable differences between groups; thus, they are not reported here.

2 We initially considered the jaw as a non-focal region, but removed it from the category because of its extensive movement during the speech event (thus providing information about the kinematics of the speech act), which distinguished it from other non-focal areas. However, we did repeat the analyses of the non-focal regions with the jaw included. This inclusion did not change the outcome for AV speech-in-noise, but it did for visual-only. In the visual-only task, there were considerably more fixations in the jaw region by the TD participants than the ASD participants (although, in an analysis of just fixations on the jaw, the difference was not statistically reliable). As a result, including jaw in the non-focal category had the effect of eliminating the statistically significant group difference in non-focal fixations. However, this obscures an interesting difference between the groups: The ASD group spent relatively more time fixating on face areas that convey less information about the kinematics of the speech articulations (e.g., the cheeks).
